# Localization and Functional Characterization of an Occipital Visual Word form Sensitive Area

**DOI:** 10.1038/s41598-018-25029-z

**Published:** 2018-04-30

**Authors:** Bo Zhang, Sheng He, Xuchu Weng

**Affiliations:** 1 Institute of Psychology, University of Chinese Academy of Sciences, Beijing, China; 2 Graduate school, University of Chinese Academy of Sciences, Beijing, China; 3 Center of Cognition and Brain Disorder, Hangzhou Normal University, Hangzhou, China; 4 State Key Laboratory of Brain and Cognitive Science, Institute of Biophysics, University of Chinese Academy of Sciences, Beijing, China; 5 Department of Psychology, University of Minnesota, Minnesota, USA; 6 School of Psychology, Xinxiang Medical University, Xinxiang, China

## Abstract

In human occipitotemporal cortex, category-specific processing for visual objects seems to involve pairs of cortical regions, often with one located in the occipital cortex and another more anteriorly. We investigated whether such an arrangement might be the case for visual word processing. In addition to the Visual Word Form Area (VWFA) located in the occipitotemporal sulcus, we observed that another region in occipital lobe with robust responses to written words (Chinese characters). The current fMRI study investigated this area’s precise location and its functional selectivity using Chinese characters and other categories of visual images (cars, chairs and insects). In all the 13 subjects we could identify a cluster of voxels near the inferior occipital gyrus or middle occipital gyrus with stronger responses to Chinese characters than scrambled objects. We tentatively label this area as the Occipital Word Form Sensitive Area (OWA). The OWA’s response amplitudes showed similar preference to written words as the VWFA, with the VWFA showing a higher degree of word selectivity, which was confirmed by the result from spatial patterns of response. These results indicate that the OWA, together with the VWFA, are critical parts of the network for processing and representing the category information for word.

## Introduction

The ability to read written words is supported by a series of complicated neural processes starting from the early visual areas to high level language centers in the brain. Neuroimaging studies have identified a ‘visual word-form area’ (VWFA), located in the left posterior Occipitotemporal Sulcus^1^, showing increased hemodynamic activation to words compared with other types of stimuli. For example, words generated stronger responses in the VWFA than line drawings^2,3^, two-tone pictures of faces and houses^4^, checkerboards^5,6^, and geometric symbols^7^.

In human occipitotemporal cortex, category-specific processing for visual objects seems to involve pairs of cortical regions, often with one located in the occipital cortex and another more anteriorly, such as the Occipital Face Area (OFA) and the Fusiform Face Area (FFA) for face processing, the Occipital Place Area (OPA) and the Parahippocampal Place Area (PPA) for place/scene processing^8–11^, or the Extrastriate Body Area (EBA) and the Fusiform Body Area (FBA) for body shape processing^12,13^. Similarly, there is some indication that a region in the occipital cortex was selectively activated when subjects viewed Chinese characters compared with scrambled version^14^. Activation clusters in the occipital cortex also were observed in some researches using alphabetic words or letters vs. baseline or faces, which showed some selectivity for orthographic stimuli^15–19^. And an adaptation paradigm study even revealed directly a word sensitive patch in occipital lobe which engaged in visual hemifield integration^20^. However, those researches did not focus on this occipital cluster or explicitly shed enough light on it on individual cortical surface. Moreover, those previous studies typically focused on the with-in category representation (i.e., words and word-like stimuli), which was far from enough to conclude that this occipital cluster was word sensitive. Further investigation about categorical representation (i.e., words vs. other objects) in this cluster could help clarify the question.Thus, is there an occipital area that is preferentially engaged for processing written words? That is the focus of current investigation. We first asked whether an occipital area could be reliably identified with selective sensitivity to words, and where it is localized anatomically and in relation to other known regions at the individual level, and further characterized this area’s functional properties in more details.

To identify and locate the putative occipital word form-sensitive area (OWA for convenience), responses to written words were contrasted to scrambled object and the resulting areas of activation in the occipital lobe were referenced to a number of known functional areas, including Lateral Occipital Complex (LOC), VWFA, human motion-selective complex (hMT+), as well as the early retinotopic areas. To further characterize the functional properties of this area, we adopted images from four different categories (Chinese character, insects, chairs, and cars) to examine the putative OWA’s response characteristics. These pictures have been used in previous studies^21–23^. For comparison, the OWA’s response properties were compared to that of the VWFA, LOC, and angular gyrus (a region thought to be important for word recognition)^24,25^. Both the conventional univariate analysis (General Linear Model) as well as a recently developed multivariate representational similarity analysis (RSA)^26^ were utilized to describe the OWA’s response selectivity and to characterize its representational structure with regard to multiple object categories, in comparison to other known ROIs.

## Results

### Location of the putative OWA

**Figure 1:**
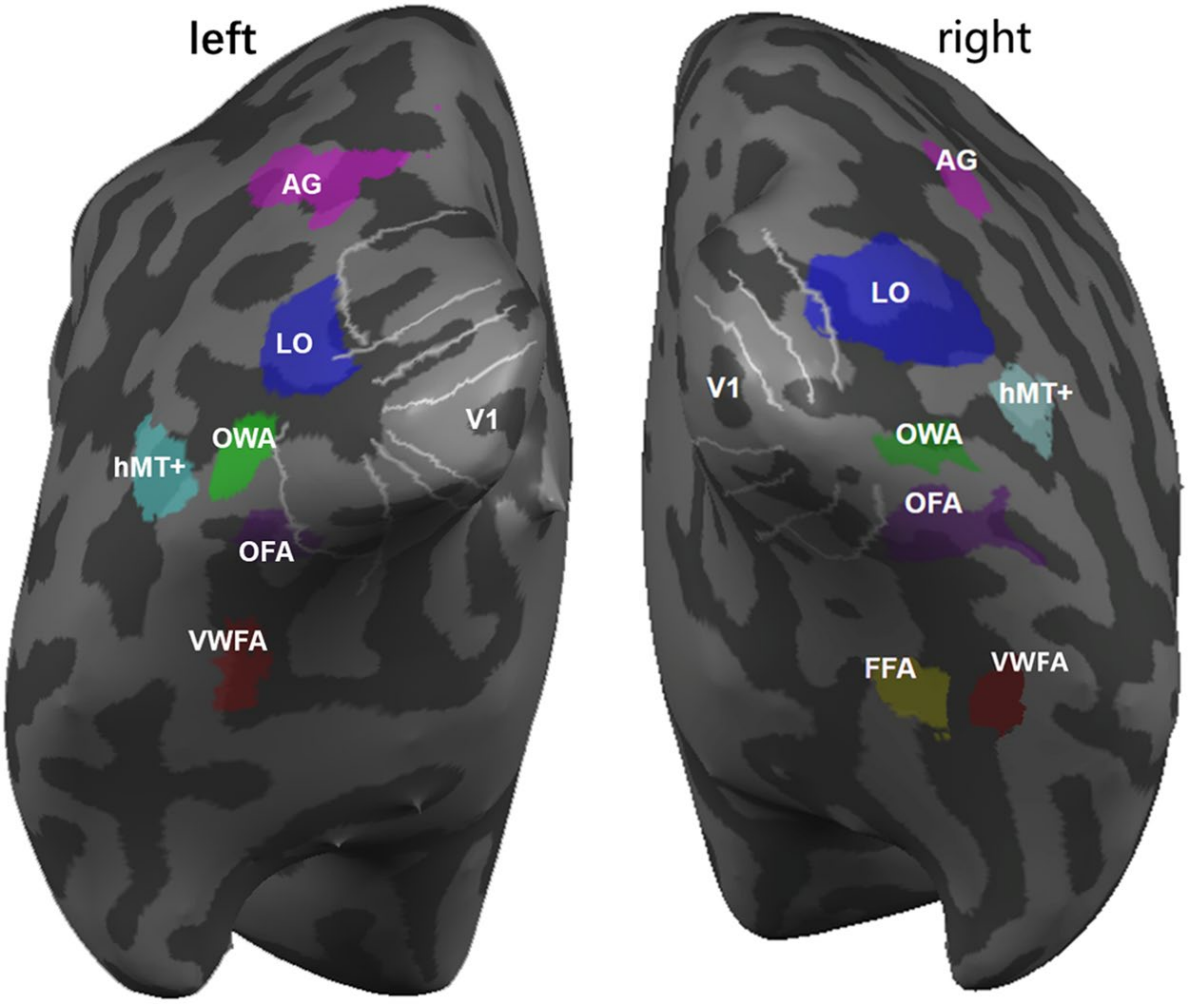
Relative spatial position of ROIs in a typical subject’s inflated brain. OWA is mainly located near inferior or middle occipital gyrus, posterior to VWFA, inferior to hMT+, more ventral to LO and anterior to the retinotopic areas. The white lines indicate the boundaries of the retinotopic areas. LO: lateral occipital complex (also named as LOC in other parts of the paper). AG: angular gyrus. hMT+:human motion-selective complex. OWA: occipital word sensitive area. OFA: occipital face area. VWFA: visual word form area. FFA: fusiform face area. V1: primary visual cortex.

Based on the contrast between Chinese characters and scrambled objects in the identification scans, a word sensitive area, putatively the OWA, could be identified in all 13 subjects in both the left and the right occipital cortex. In terms of its anatomical site, in the 13 subjects the putative OWA was located near the inferior occipital gyrus (8 subjects), the middle occipital gyrus (4 subjects), or the posterior part of inferior temporal sulcus (1 subject). In normalized Talairach space, the coordinates of the OWA averaged across subjects are [−41, −76, −6] in the left hemisphere and [39, −73, −6] in the right hemisphere (see Table 1 for individual Talairach coordinates). Since we identified the VWFA, LOC, and FFA individually in all the subjects and early retinotopic visual areas (V1/V2/V3/hV4) and hMT+ in 5 subjects, the location of the OWA could be described in spatial relationships to these known cortical regions. As shown in Fig. 1, the OWA is posterior to the VWFA, inferior to hMT+, more ventral to LOC, and anterior to the retinotopic areas. On average, the distance between the OWA and VWFA is 22.9 mm.

**Table 1:** Talairach coordinates of center and cluster sizes of ROIs for each subject and mean values across subjects (unit of SD: mm).

ID	LOWA				ROWA			
	X	Y	Z	Volumn (mm^3^)	X	Y	Z	Volumn (mm^3^)
S1	−42	−82	−6	703	45	−50	−4	399
S2	−43	−72	−18	2077	35	−78	−16	649
S3	−39	−71	−5	1022	41	−74	−6	767
S4	−52	−68	−2	1326	38	−78	−6	613
S5	−41	−83	7	1124	40	−74	3	838
S6	−57	−57	−3	485	37	−76	−5	927
S7	−38	−74	−6	1494	43	−74	−2	1250
S8	−41	−79	−7	1176	41	−68	−7	1462
S9	−35	−74	−8	627	40	−79	−6	541
S10	−45	−77	−6	636	45	−70	−4	1541
S11	−49	−64	−9	996	37	−72	−11	250
S12	−38	−82	−7	730	37	−78	−7	1801
S13	−31	−84	12	709	33	−80	1	520
Mean(SD)	−41(4)	−76(5)	−6(5)	1035	39(3)	−73(8)	−6(5)	889
**ID**	**LVWFA**				**RVWFA**			
	**X**	**Y**	**Z**	**Volumn (mm** ^**3**^ **)**	**X**	**Y**	**Z**	**Volumn (mm** ^**3**^ **)**
S1	−50	−49	−8	1015	43	−38	−17	333
S2	−40	−62	−18	709	N	N	N	N
S3	−38	−44	−16	649	44	−51	−8	546
S4	−48	−60	−12	724	44	−54	−14	341
S5	−46	−64	−7	641	44	−53	1	577
S6	−44	−59	−12	694	39	−64	−13	564
S7	−45	−42	−14	817	N	N	N	N
S8	−48	−52	−10	1201	40	−45	−15	647
S9	−47	−56	−9	520	49	−50	−9	304
S10	−51	−50	−13	660	N	N	N	N
S11	−46	−61	−8	499	37	−54	−14	534
S12	−49	−52	−14	728	37	−69	−13	820
S13	−35	−45	−7	327	30	−41	−12	659
Mean(SD)	−45(5)	−54(7)	−11(4)	694	41(5)	−52(9)	−11(5)	533

### Functional Selectivity to Words of the OWA

Having reliably identified and localized the putative OWA, we next characterized functional properties of this region, in particular its degree of word selectivity, by comparing with responses of the VWFA and other visual regions. We used univariate analysis to examine these ROIs’ activation strength to different stimulus categories and used multivariate analysis to examine these ROIs’ ability to discriminate different stimulus categories.

#### Word selectivity in OWA and other ROIs measured in response amplitude

**Figure 2:**
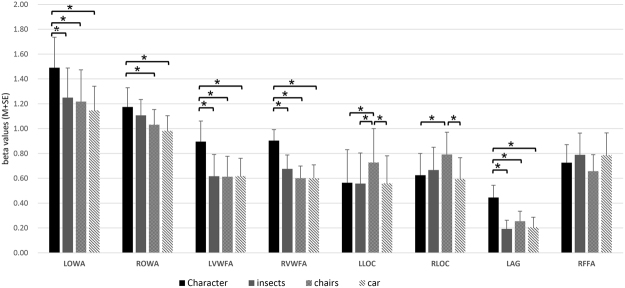
Summary of response amplitudes in the ROIs across subjects. The error bars denote the standard error calculated across subjects. The “*” indicates the mean difference is significant at the 0.05 level in the multiple comparisons and Sidak correction is applied. OWA shows similar selectivity pattern to character (words) as the VWFA. LOC, as control ROI, response to chairs strongest. (N = 10 for all the ROIs except that N = 8 for RVWFA, N: number of subjects) (the prefix “L” or “R” before each ROI name denotes left or right).

As described in the previous section, the OWA responds robustly to words. How selective is its response to words, and how does its word selectivity compare with other ROIs in the occipito-temporal cortex? To answer this question, we compared the response magnitudes to four types of images (words, insects, chairs, cars) in bilateral OWA, bilateral VWFA, bilateral LOC, left angular gyrus, and right FFA. Words generated strongest responses among the four types of images in OWA, VWFA, and AG. In contrast, LOC responded a little more strongly to chairs, and FFA showed no preferential response to any of the four types of images. As the number of different ROIs is not equal (N = 10 for all the ROIs except that N = 8 for RVWFA), which case was not suitable for two-way ANOVA, so for each ROI we just first performed single-factor repeated measures ANOVA and then pairwise comparisons for the four categories. As for the problem of multiple comparisons, Sidak correction was adopted to decrease the likelihood of type I error in the post hoc. The results showed that the words generated significant higher signal than insects (*p* = 0.003), chairs (*p* = 0.002) and cars (*p* = 0.005) in the left OWA (*F*(3, 27) = 9.453, *p* < 0.001), and the response pattern was very similar in the left VWFA (*F*(3, 27) = 8.679, *p* < 0.001), the right VWFA (*F*(3, 21) = 8.13, *p* < 0.001), and the left AG (*F*(3, 27) = 11.506, *p* < 0.001) (Fig. 2). In right OWA, the words generated significant higher signal than chairs (*p* = 0.024) and cars (*p* = 0.015), but not insects (*p* = 0.46) (*F* (3, 27) = 6.088, *p* = 0.003). However, bilateral LOC, as control ROIs, responded strongest to chairs (almost all *p* < 0.05, compared with words, insects, cars) (left: *F*(3, 27) = 5.047, *p* = 0.007; right: *F*(3, 27) = 6.788, *p* = 0.002); and no significant difference was found between the response magnitudes of the categories in right FFA (*F* (3, 27) = 1.211, *p* = 0.32).

**Figure 3:**
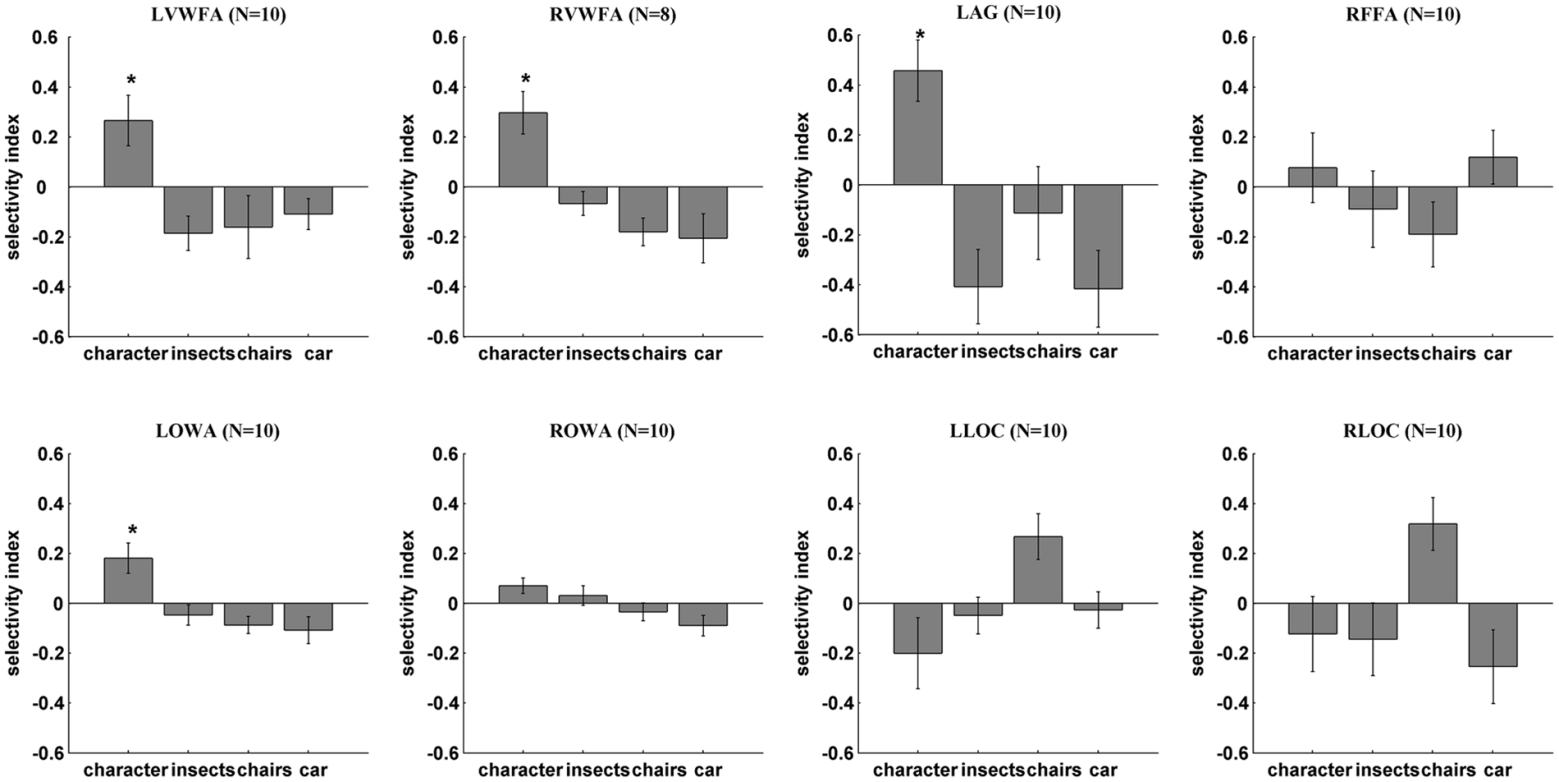
Selectivity index (SI) based on beta values from univariate analysis. Positive value indicates the degree of selectivity to the corresponding category relative to other categories, negative value indicates the degree of unpreference to the category relative to other categories. The LAG gains the maximum mean SI to character, followed which are LVWFA and LOWA. However, the difference of SI to character between LOWA and LVWFA or LAG are neither significant .The error bars denote the standard error calculated across subjects. The “*” denotes the character –SI were significantly larger than 0. (N = 10 for all the ROIs except that N = 8 for RVWFA. N: number of subjects) (the prefix “L” or “R” before each ROI name denotes left or right).

In order to quantify the degree of selectivity to words across different ROIs, A Selectivity Index (SI) was calculated for each stimulus category in each ROI (Fig. 3).

1 $${\rm{Selectivity}}\,{\rm{Index}}=\frac{A-B}{\sqrt{{{\rm{A}}}^{2}+{{\rm{B}}}^{2}}}$$

where A represents the beta value for the stimulus category under consideration, B represents the mean of the beta values for the other 3 stimulus categories. Statistical analysis was then performed on the selectivity indices for each ROI. Results based on one sample two-tailed T test show that among the word-related ROIs, the SIs for character in the following ROIs were significantly above 0: LOWA (*t*(9) = 2.96, *p* = 0.016), LVWFA (*t*(9) = 2.62, *p* = 0.027), RVWFA (*t*(7) = 3.49, *p* = 0.01) and LAG (*t*(9) = 3.72, *p* = 0.005), while the SI for character in the ROWA was marginally above 0 (*t*(9) = 2.25, *p* = 0.052). However, the difference of SI to character between LOWA and LVWFA was not significant (*t*(9) = 0.73, *p* = 0.48).

#### Word selectivity in OWA and other ROIs measured in spatial patterns of response

**Figure 4:**
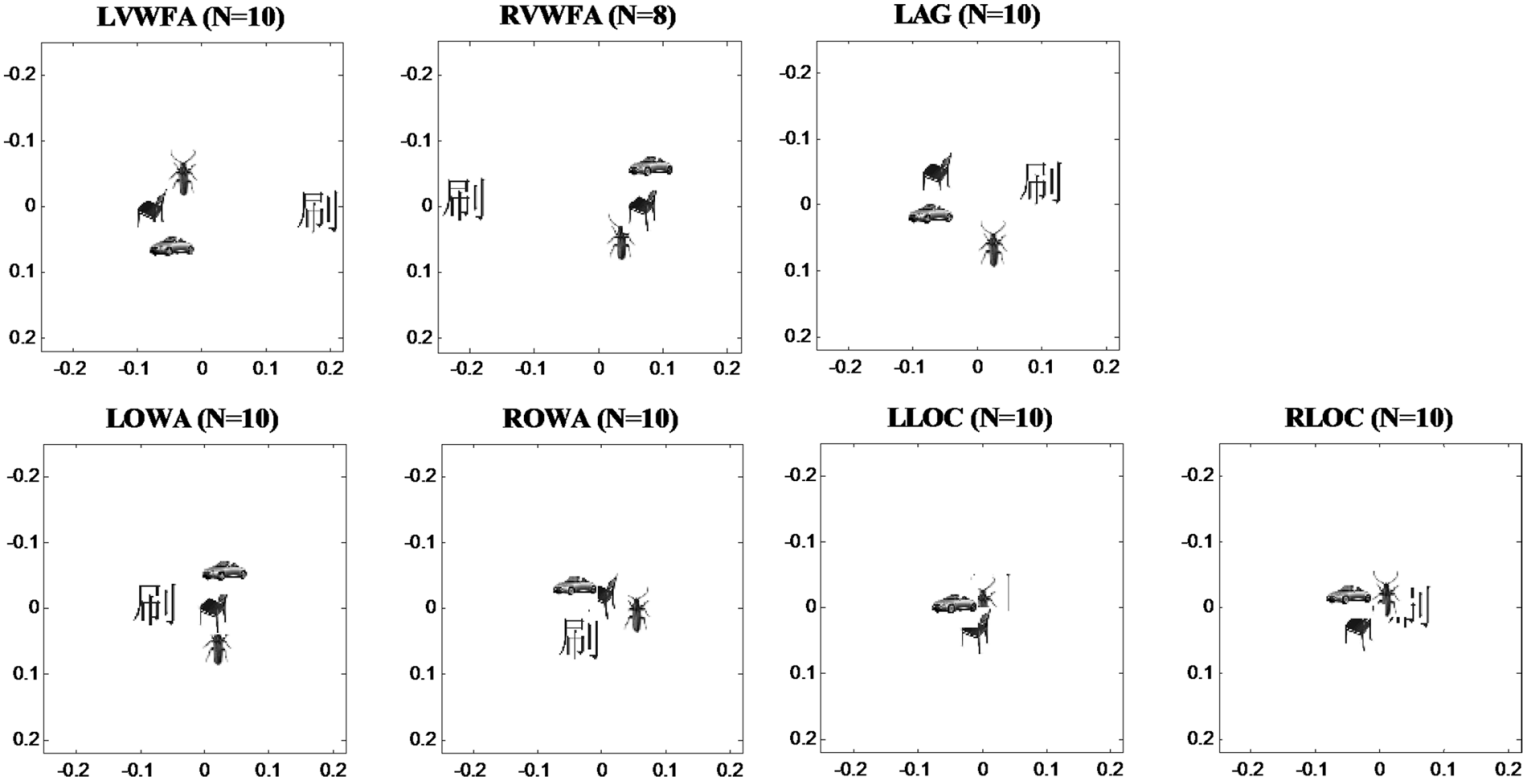
Multi-dimensional scaling. (Dissimilarity: 1- Pearson’r, criterion: stress) for each ROI, stimulus categories with similar activation patterns are placed close together. The distance between stimuli reflects their representational dissimilarity. Left OWA shows a similar representation structure for the four categories as the VWFA, comparing with LOC. But VWFA represents character further from other categories comparing with OWA.

Univariate analysis of neural responses aggregates activation across the whole region at the cost of ignoring the rich pattern information within each ROI. In recent years, multivariate pattern analyses have gained popularity, which are capable of extracting the spatial pattern of activation information of relevant cortical areas^22,27–29^. Hence, we also analyzed the spatial patterns of response to each stimulus condition within the pre-defined ROIs, and calculated the pairwise Pearson correlations between the spatial patterns for all stimulus conditions. Thus for each ROI, we obtained a correlation coefficients matrix (i.e., representation similarity matrix) which shows the relationship between the spatial activation patterns for every pair of stimulus categories. To visualize the representational relationships between different stimulus categories, Multi-dimensional scaling (MDS) was performed and the results displayed in Fig. 4. Variations of the data explained by the two axes for each ROI were: 97% in LVWFA, 99% in RVWFA, 98% in LAG, 97% in LOWA, 98% in ROWA, 96% in LLOC, 98% in RLOC, which indicated the model fitted quite well. Intuitively in the MDS plots, stimulus categories with similar activation patterns are placed close together. MDS results (Fig. 4) showed that left OWA had a similar representation structure for the four categories of stimuli as the VWFA, comparing with LOC. Representation structure here means the relative position to each other for the categories (e.g. car, chairs and insects fall into a vertical order, while character lies on their left or right side horizontally, in LOWA and VWFA.) as axes represent potential meaningful dimensions respectively. However, the categories fall together around in LOC’s space. Particularly, VWFA represented character further from other categories comparing with OWA, suggesting that VWFA probably had a better ability to differentiate character from other categories than OWA.

**Figure 5:**
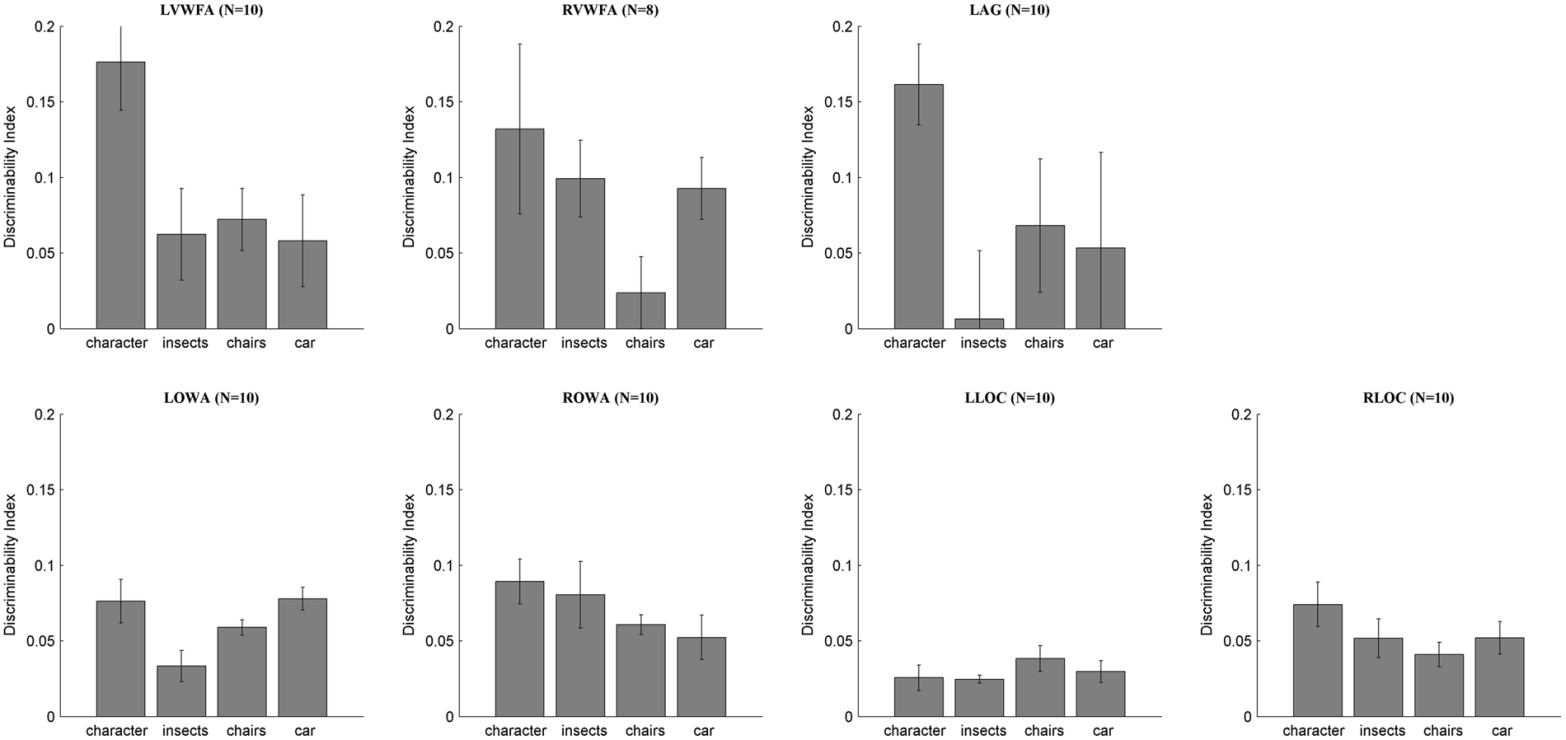
Discriminability index (DI) based on RSA from multivariate analysis. The DI reflects the ROI’s ability of discriminate one category from others, though each ROI could represent multiple categories. LOWA gained significantly less DI to character and was less specific to character comparing with LVWFA. The error bars denote the standard error calculated across subjects. (N: number of subjects) (the prefix “L” or “R” before each ROI name denotes left or right).

To further quantify the category selectivity of these different regions, we calculated the discriminability index (DI) based on the Representation Similarity Analysis (RSA) (Fig. 5). Firstly, the experiment data were split into two subsets (even and odd runs). For each subsets and for every ROI separately, we then extracted activation patterns for each category, corresponding to a list containing the responses (parameter estimates) of all voxels in the ROI. Then the values of the odd runs were correlated against those of the even runs, resulting in an asymmetrical 4 × 4 correlation matrix. These correlations represent the similarity between the activation pattern for a stimulus in the first subset of the data and the activation pattern for a (different or same) stimulus in the second subset of the data. These matrices were then made symmetrical by replacing every cell (i, j) by the mean of (i, j) and (j, i). Lastly, discriminability indices to the 4 stimulus conditions for each ROI of each subject were computed as:

2 $${\rm{Discriminability}}\,{\rm{Index}}=\frac{A-B}{\sqrt{{{\rm{A}}}^{2}+{{\rm{B}}}^{2}}}$$

where A indicates the Pearson correlation coefficient of within category for a specific condition (i.e. the value of cell (i, i) in the matrices), B indicates the Pearson correlation coefficient of between category for that specific condition (i.e. the mean of the other three elements in the same row with cell (i, i)). Those values were entered in a repeated measures ANOVA using ROI (except the RVWFA which had a smaller N) and category as within-subject factors. Statistical results revealed significant main effects of category (*F*(3, 27) = 4.337, *p* = 0.012) and ROI (*F*(5, 45) = 3.086, *p* = 0.018), and a significant interaction of ROI and category (*F*(15, 135) = 1.93, *p* = 0.025). Pairwise comparisons were further explored in ROIs and sidak method was adopted to correct the p value. In LOWA, the DI for character was larger than that for insects (*t*(9) = 4.3, *p* = 0.002), but with no difference than car (*t*(9) = 0.03, *p* = 0.98) or chairs (*t*(9) = 1.58, *p* = 0.15). In ROWA the DI for character was larger than that for chairs (*t*(9) = 3.21, *p* = 0.011) and car (*t*(9) = 2.41, *p* = 0.04), but not than insects (*p* = 0.28). In LVWFA, the DI for character was larger than that for all the other categories (*t*(9) = 3.21, *p* = 0.011 for insects; *t*(9) = 4.31, *p* = 0.002 for chairs; *t*(9) = 4.04, *p* = 0.003 for car). In RVWFA, the DI for character was only larger than chairs significantly (*t*(7) = 2.72, *p* = 0.03). In LAG, the DI for character was larger than that for insects (*t*(9) = 2.84, *p* = 0.02) and chairs (*t*(9) = 2.33, *p* = 0.052), but not than car (*t*(9) = 1.78, *p* = 0.11). However, no significant difference was detected when comparing the DI for chairs with that for any other categories in bilateral LOC. Additionally and more importantly, the DIs for character across ROIs were pairwise compared (particularly that LOWA < LVWFA (*t*(9) = 3.85, *p* = 0.004) and LOWA < LAG (*t*(9) = 2.97, *p* = 0.016)),which indicated that LVWFA had a better ability to differentiate character from other categories than LOWA and confirmed the result from MDS.

And according to the mean of the DIs for character, the rank of the word-related ROIs’ selectivity for words were: LVWFA > LAG > RVWFA > ROWA > LOWA.

## Discussion

In this study, we identified a region in the occipital lobe with robust responses to written words (Chinese character) compared with other control stimuli. We tentatively label this region as the Occipital Word-Form Sensitive Area (OWA). We explicitly and carefully mapped the location of the OWA on cortical surface, and referencing its location to both anatomical and functional landmarks at the individual level. Our study provided a finer precision for the location of occipital word area than the previously published studies^19,30^. Results showed that the OWA has similar functional properties as the VWFA, but with significantly less degree of specialization for words. Moreover, the OWA is left-lateralized in terms of its word-response robustness and word-selectivity. The study extends earlier findings of the functional areas in ventral occipitotemporal cortex and complements the understanding of the network for representing the category information for word.

### Relationship between the OWA, VWFA, and AG

Since the VWFA has been much more extensively studied, it could serve as an important reference for the OWA identified in this study. In terms of the spatial locations, the OWA is located in the middle or inferior occipital gyrus while the VWFA is usually located more anteriorly in or near the occipital-temporal sulcus. The Talairach coordinates of the VWFA have been reported as [−43 −56 −16]^31^ transformed from MNI coordinates using mni2tal function in matlab and [−43 −52 −12]^1^, consistent with the coordinates [−45 −54 −11] identified for VWFA in the current study. The left OWA identified in this study at [−41 −76 −6] is about 2 cm posterior and slightly superior to the VWFA (see Fig. 1). In terms of the functional specificity, VWFA has strong response preference to words as shown in many studies^5,21,32,33^ and in the current study; left OWA also has stronger responses to words than other categories (Fig. 3). Thus the univariate results based on response levels demonstrate that the left OWA is robustly and selectively engaged in processing words. On the other hand, the multivariate analysis provided a somewhat different picture – while the multivoxel patterns of activity in VWFA are highly specific for words, the OWA’s multivoxel patterns showed much weaker specificity for words (Figs 4, 5). An area that is strongly engaged in processing words (higher response amplitude) does not necessarily mean that this area will have a unique response pattern for words. In addition to the OWA and the VWFA, we also included the angular gyrus (AG) as a reference in this study. The left AG, a region known to be involved in certain cognitive tasks such as reading and spatial attention, not surprisingly showed very strong response to characters compared with other stimulus categories (Fig. 3). Indeed, the left AG had the highest mean SI to characters, followed by the left VWFA and left OWA, though the SI difference among them was not significant. Our results are consistent with the idea that a hierarchical organization of low-to-high orthographic sensitivity that progresses anteriorly along the ventral stream^15,34–36^, and further suggest that the VWFA engages in the processing of orthography information at the global level while the OWA may be involved in the processing of character components at a more local level. Our data also indicate that the angular gyrus may further process the phonetic and semantic information of the characters^37^, and possibly exert top-down modulation on information flow from the OWA to higher areas^38^. More specifically designed experiment on the functional role of AG would be required to test this possibility in the future. Future studies will need to manipulate word components (e.g., radicals of Chinese characters or pseudo-characters) in order to address the next question whether OWA and VWFA form a hierarchy to encode Chinese characters at different levels of complexity.

### Relationship between word sensitive regions and face sensitive regions

Compared to another well identified occipital region potentially at a similar processing stage in the visual object processing hierarchy, the OFA at [−20 −90 −12]^39^, the OWA appears to be more posterior and superior. It was suggested that face-selective patches were alternated by limb-selective patches, and superior to IOG-face patch, an IOG/MOG-word patch seemed reasonable^40,41^, which is supported by our result. Other evidence showed that characters-sensitive patch near IOS was superior to face-sensitive patch near IOG^42^. However, a more striking match between the location of the OFA and OWA is found by Strother *et al*.^20^. This discrepancy is probably due to the different contrast method adopted in Strother *et al*. study and others (including the current study) to define the ROI. In our study, “Chinese characters > scrambled object” (p < 0.005, cluster-level FWE corrected) was adopted to obtain OWA [Talairach: −41, −76, −6] in the left hemisphere. In contrast, Strother *et al*. adopted “Different condition > the Same condition” (q(FDR) < 0 .05) to obtain OWFA [Talairach: −38, −80, −10] in the left hemisphere, where is symmetric to the known location of the OFA in the right hemisphere. A newly published electrocorticography study revealed that in the occipitotemporal cortex there appeared to be three letterstring-selective zones: one located anterior to the anterior end of the mid-fusiform sulcus and near the collateral sulcus, another in the anterior portion of the mid-fusiform sulcus just behind the anterior face zone, and the third located in the posterior portion of the mid-fusiform sulcus^43^. We indeed observed activation clusters near the collateral sulcus besides the VWFA and OWA in a subset of subjects, but this region is not as robust as the VWFA or OWA. Taken together, the distribution of the face and word sensitive regions appeared to be alternated and partially overlapped, and the OWA and OFA are likely performing similar types of computations for written words and faces respectively.

### OWA for written words from different language systems

Many previous studies on the processing of written words show that the location and the properties of the VWFA is quite consistent across different populations, different language systems, and robust to the different contrasts adopted^21,32,44–46^. We believe that the OWA investigated in the current study using Chinese characters is also invariant to the types of scripts used. A previous study used English words and letter strings as stimuli, revealing what the authors called a “letter-form” area posterior to the VWFA at the Talairach coordinates of [−40, −78, −11]^16^, which is very close to the OWA [−41 −76 −6] localized in the current study. The putative ‘letter-form’ area was defined by the contrast of consonant strings versus false fonts. In another study also using Chinese characters, contrasting real-characters versus their scrambled versions revealed character sensitive voxel clusters^14^. And the activation clusters includes the fusiform character area (FCA) in the middle fusiform gyrus, the middle and the inferior occipital gyri (MOG and IOG, respectively), the lateral occipital character area (LOCA) and the occipitoparietal character area (OPCA). The MOG and IOG clusters were consistent with the location of the OWA in the current study. Additionally, the MOG/IOG were found to be activated both by Chinese character and alphabetic words with the activation likelihood estimation (ALE) method in a meta-analysis literature^47^. Clearly more confirmative studies are needed, though, but the results from the few studies relevant to the OWA suggest that it should be identifiable across different writing systems.

## Methods

### Subjects

Thirteen native Chinese speakers (seven females) participated the following OWA identification and localization scan, and among them 11 subjects completed the additional study characterizing the functional properties of the OWA. And then detailed retinotopic maps and hMT+ were obtained from five of these subjects. All subjects were between 21–32 years old, right-handed, and had normal or corrected-to-normal vision. All experiments and procedures were approved by the Institutional Review Board (IRB) of the Center of Cognition and Brain Disorder at the Hangzhou Normal University. All subjects were given written, informed consent before taking part in the experiment. And methods were carried out in accordance with the approved guidelines.

### Experimental stimuli, designs and procedures

Stimulus presentation was controlled using E-prime (Psychology Software Tools, Pittsburgh, USA). A Barco 6400i LCD projector (resolution 1024 × 768, refresh rate 75 Hz) was used to project the stimuli on a screen positioned approximately 40 cm from subjects’ eyes. Subjects viewed the stimuli via a mirror attached to the head-coil.

#### OWA identification and localization

**Figure 6:**
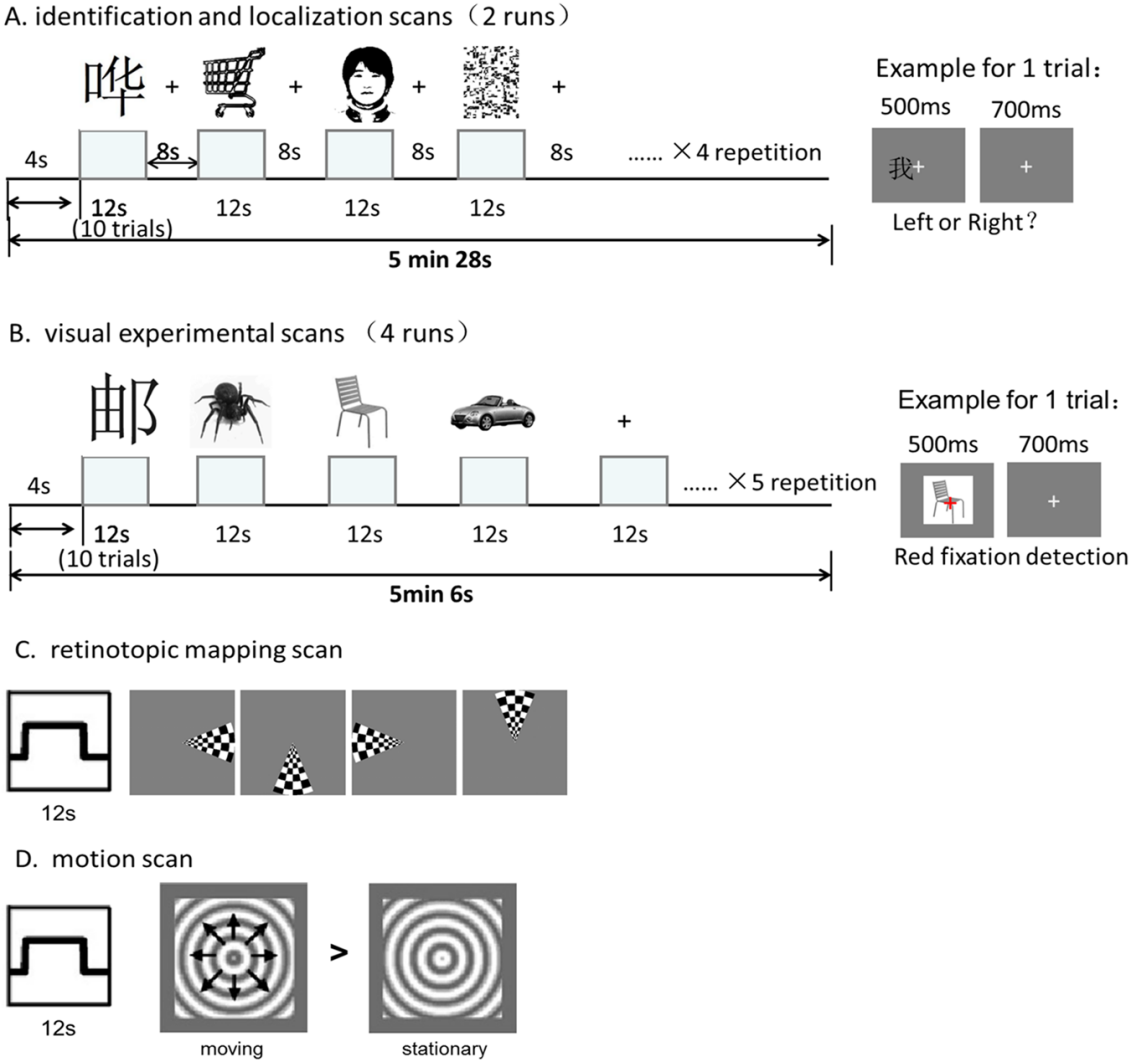
Experiment design and procedures.

This part included a “localizer scan”, which was used to identify word-sensitive Regions of Interest (ROIs) and provided a basis for subsequent ROI based analyses. Different stimuli and tasks were used for this ROI localization scan and the subsequent scans to characterize the properties of the identified ROI, so as to ensure the independence of the data and avoid the circularity problem^48^. Subjects participated in 2 runs of this scan while they viewed two-toned images of faces, Chinese characters, common objects, and scrambled objects in 12-s blocks interleaved by 8-s fixation (Fig. 6A). The 4 different block conditions with 4 repetitions were randomly arranged in each run. A 4-s blank screen was presented at the beginning of each run for the dummy scan and a 4-s blank screen was presented at the end of each run. So the total time is 328 s for a run. Ten stimuli per 12-s block were visually presented, each for 500 ms with an inter-stimulus interval of 700 ms. The center of each picture was slightly shifted from the fixation point, and subjects were asked to perform a “right-left judgment” task, on whether the stimulus was to the right or the left relative to the fixation point. All stimuli appeared pseudo-randomly in a rectangular area with a visual angle of 4.6 degrees in width and 6 degrees in height, against a gray background.

#### OWA characterization

Four types of grey-scaled image stimuli including Chinese characters, insects, cars and chairs were adopted in block design to investigate the predefined ROIs’ sensitivity in 4 visual experiment runs (Fig. 6B). Insects represent a typical category for living or animated things, cars and chairs are commonly used for object processing. We used these different categories as controls. In each run, there are 20 stimuli blocks (5 repetitions for each condition) and 5 rest blocks, randomly arranged. Each block lasted 12 s. A 4-s blank screen was presented at the beginning of each run for the dummy scans and a 2-s blank screen was presented at the end of each run. So the total time is 306 s for a run. Ten stimuli per stimuli block were visually presented on the center of the screen, each for 500 ms with an inter-stimulus interval of 700 ms. During the whole course of a run, a fixation cross always appeared in the center of the screen. And subjects were required to perform a fixation color change detection task: press the button once the fixation cross turned red from white. All stimuli were displayed on a uniform gray background with a width of 2.6° visual angle.

#### Mapping the retinotopic areas and hMT+

Conventional wedge sections of a high-contrast, flashing checkerboard pattern were used^49,50^. The wedge subtended 45° polar angle. The maximum stimulus radius was 8° of visual angle. They followed a periodic pattern and complete a full cycle in 48 s with a total of 6 cycles per scanning run. In each cycle, the flickering wedge was presented for 12 s at each of four meridian locations (right, lower. left, upper) clockwise sequentially around the fixation point in the center of the visual field (Fig. 6C). At regular intervals between each cycle, the wedge was removed and subjects viewed a mean luminance gray background with the fixation, which lasted 12 s. Subjects performed a fixation task during which they responded by button press when the fixation point changed color. Two runs were employed. For hMT+ localization, subjects viewed 6 alternations of 16-s blocks of low contrast expanding and contracting concentric gratings and 16-s blocks of identical stationary gratings while fixating (Fig. 6D).

### Data acquisition

Anatomical and functional imaging data were acquired on a 3T MR750 General Electrical scanner using an 8-channel head coil. Subjects’ head motion was minimized by placing padding around the head. Functional MR data were acquired using single-shot gradient-recalled echo-planar imaging. There were 42 axial slices with full-brain coverage, and the slice thickness was 3 mm with no gap. Slices were acquired in an interleaved order. The other MR parameters were Field Of View (FOV) = 192 mm, 64 × 64 matrix, TR = 2000 ms, TE = 30 ms, Flip angle = 75°. A high-resolution anatomical volume of the whole brain was acquired using a T1-weighted SPGR pulse sequence (TR = 9 ms, flip angle = 45°, FOV = 220 mm, resolution of 1 × 1 × 1 mm^3^).

### Data analysis

All data were analyzed with Brainvoyager software (Brain Innovation) and custom-built Matlab codes. Further statistical analyses were performed using SPSS. Anatomical volumes were segmented into gray and white matter and from this segmentation we reconstructed the cortical surface for subject. Each subject’s functional data were aligned to their high-resolution anatomical volume, enabling us to compare data across sessions and to visualize activations on the inflated cortical surface. Preprocessing involved the following steps: correction for differences in acquisition time, motion correction (and extraction of motion parameters), realignment of the functional scans to the anatomical volume, normalization to Talairach space, and unsmoothed. Voxel size was resampled to 3 mm isotropic.

#### Identification of word sensitive areas and localization of reference ROIs

Statistic maps of the brain were computed by performing general linear model multiple regression tests in data from identification scan and motion scan. The data from the identification scan were used to determine word-sensitive (VWFA, OWA) and object-selective (i.e., the lateral occipital complex, LOC) areas in individual subjects. As we defined the ROIs individually rather than basing on group-level analysis, the response amplitude results would be less degraded due to mis-registrations of ROIs across subjects^51^. The VWFA was defined as region constrained near the occipitotemporal sulcus in which Chinese characters generated higher activity than scrambled object (*p* < 0.005, cluster-level FWE corrected), similar contrast was employed by previous researches^31,51,52^. Also, the same contrast was used to define the OWA (*p* < 0.005, cluster-level FWE corrected, anatomically constrained in the occipital lobe) and an area sensitive to Chinese characters in Angular Gyrus (AG, *p* < 0.005, cluster-level FWE corrected) which has long considered to be involved in reading^25^. As these ROIs’ functional selectivity would be independently characterized next, we did not apply a strong “selectivity” bias at the stage of identifying the ROIs. Hence the ROIs were defined by contrasting words with scrambled objects. LOC was defined as continuous voxels that showed significantly greater activation (*p* < 0.001, cluster-level FWE corrected) for objects compared to scrambled objects, in lateral occipital regions^53^. Additionally, the fusiform face area (FFA) and the occipital face area (OFA) were identified as continuous voxels that showed significantly greater activation (*p* < 0.0001, cluster-level FWE corrected) for face compared to Chinese characters, in the fusiform gyrus and the occipital lobe respectively.

#### Retinotopic mapping and hMT+ localization

The hMT+ was defined as a cluster in the posterior inferior temporal sulcus (ITS) that responded more strongly to moving than stationary gratings (*p* < 0.002, cluster-level corrected)^54,55^. For the data from retinotopic mapping scan, activation maps of the brain were computed by performing cross-correlation analysis to obtain the phase-encoded retinotopic map^56,57^, the retinotopic areas in early visual cortex were delineated individually, which together with the LOC and the hMT+, served as spatial references for OWA.

#### Univariate analysis for charactering properties of the word sensitive areas

Due to the large head motion problem in the OWA characterization scan, one subject was excluded from further analysis. In the remaining 10 subjects, time course in all the runs were normalized according to their fixation blocks and all runs were combined for further analysis. For each pre-defined ROI, a general linear model with totally 11 regressors (with 5 independent variables corresponding to the 4 stimuli categories and 1 fixation condition, in addition to 6 covariates corresponding to the movement parameters for rotations and translations) was adopted to obtain parameter estimates (beta values) for each stimulus condition at the whole ROI level for each subject separately. For each ROI, statistical analysis was then performed on the averaged beta values to different stimulus conditions across subjects.

#### Multivariate analysis for charactering properties of the word sensitive areas

For each pre-defined ROI, a general linear model with totally 11 regressors (with 5 independent variables corresponding to the 4 stimuli categories and 1 fixation condition, in addition to 6 covariates corresponding to the movement parameters for rotations and translations) was adopted to obtain parameter estimates (beta values) for each stimulus condition in each voxel for each of the 10 subjects separately. We then calculated pairwise Pearson correlations for the beta value patterns between all the four categorical conditions similarly to previously published methods^28^. These correlations represented the similarity between the activation pattern for a stimulus category and the activation pattern for a (different or same) stimulus category. So for each ROI, we calculated a 4 × 4 symmetrical Pearson correlation coefficients matrix from the beta patterns, and then we converted it into a dissimilarity matrix, by subtracting each cell from 1. This dissimilarity matrix was then reduced to a two-dimensional space for visualization through non-classical Multi-dimensional scaling (MDS) based on PROXSCAL algorithms within SPSS 20.0. Each category was positioned on the two-dimensional plane, in which the distance between any pair of categories reflects the correlation between their response patterns (the higher the correlation the closer the distance).The reason of choosing two dimension was that the stimuli categories could be arranged from two aspects: the size in real world and abstractness level. Meanwhile MDS here visualizes the level of similarities of the categories without assuming clear internal structure. Lastly, the signal-to-noise ratio (SNR) values from rest blocks of the experiment runs in all ROIs were not significantly different from each other (figure in Supplementary Information).

### Data availability statement

The data that support the findings of this study are available from the authors upon reasonable request.

## Electronic supplementary material

supplementary information
