# Supplementary material for: Localization and Functional Characterization of an Occipital Visual Word form Sensitive Area
Source: Sci Rep. 2018 Apr 30;8:6723. doi: 10.1038/s41598-018-25029-z (PMC5928127; doi:10.1038/s41598-018-25029-z)
Supplement: Supplementary file 1 — supplementary information [file 41598_2018_25029_MOESM1_ESM.pdf]

Localization and Functional Characterization of an Occipital Visual Word Form Sensitive Area

Bo Zhang [1, 2, 6], Sheng He [4, 5*], Xuchu Weng [1, 3*]

[1]Institute of Psychology, University of Chinese Academy of Sciences, Beijing, China;
[2]Graduate school ,University of Chinese Academy of Sciences, Beijing, China;
[3]Center of Cognition and Brain Disorder, Hangzhou Normal University, Hangzhou, China;
[4]State Key Laboratory of Brain and Cognitive Science, Institute of Biophysics, University of Chinese Academy of Sciences, Beijing, China;
[5]Department of Psychology, University of Minnesota, Minnesota, US
[6]school of psychology, Xinxiang Medical University, Xinxiang, China

* Correspondence: sheng@umn.edu (Sheng He), wengxc@psych.ac.cn (Xuchu Weng)

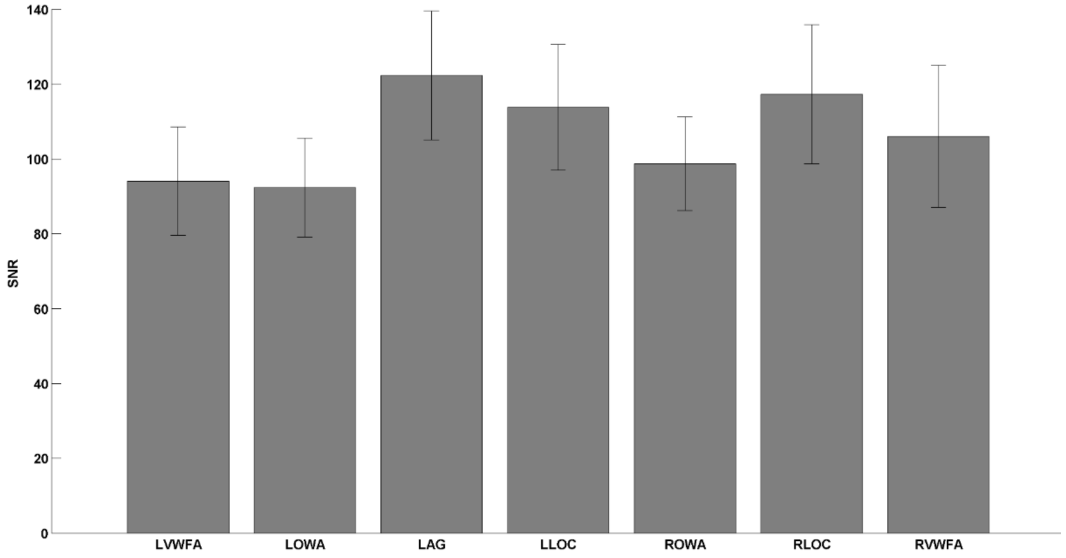

The signal-to-noise ratio (SNR) in each ROI. (N=10). The error bars denote the standard error calculated across subjects. The SNR was calculated as follows: (1) For those fixation blocks（rest blocks）in all runs , we calculated the mean and standard deviation (SD) in every voxel over time. SNR was then calculated on a per-voxel basis by dividing the mean signal by the SD calculated across all time points; (2) For each subject, a combined SNR map was obtained by averaging the SNR map for each run; (3) The ROI masks were used to extract the SNR in each ROI by taking the mean of the SNR of the voxels within the ROI; (4) The SNR for each ROI was averaged across the subjects. Repeated measures ANOVA found no significant difference across the ROIs($F(6，54)=1.48$, $p=0.21$).Therefore, the functional differences between ROIs might not be attributable to systematic differences in their SNR.
